# Retrospective Evaluation of Implants Placed in Iliac Crest Autografts and Pristine Bone

**DOI:** 10.3390/jcm11051367

**Published:** 2022-03-02

**Authors:** Florian Beck, Georg Watzak, Stefan Lettner, André Gahleitner, Reinhard Gruber, Gabriella Dvorak, Christian Ulm

**Affiliations:** 1 Division of Oral Surgery, University Clinic of Dentistry, Medical University of Vienna, 1090 Vienna, Austria; georg.watzak@meduniwien.ac.at (G.W.); christian.ulm@meduniwien.ac.at (C.U.); 2 Core Facility Hard Tissue Research and Biomaterial Research, Karl Donath Laboratory, University Clinic of Dentistry, Medical University of Vienna, 1090 Vienna, Austria; stefan.lettner@meduniwien.ac.at; 3 Austrian Cluster for Tissue Regeneration, Ludwig Boltzmann Institute for Experimental and Clinical Traumatology, 1200 Vienna, Austria; 4 Department of Biomedical Imaging and Image-Guided Therapy, Division of Neuroradiology and Musculoskeletal Radiology, Medical University of Vienna, 1090 Vienna, Austria; andre.gahleitner@meduniwien.ac.at; 5 Department of Oral Biology, University Clinic of Dentistry, Medical University of Vienna, 1090 Vienna, Austria; 6 Department of Periodontology, School of Dental Medicine, University of Bern, 3012 Bern, Switzerland; 7 Division of Conservative Dentistry and Periodontology, University Clinic of Dentistry, Medical University of Vienna, 1090 Vienna, Austria; gabriella.dvorak@meduniwien.ac.at

**Keywords:** mandible, autologous transplants, atrophy, dental implants, iliac crest bone, implant-supported denture

## Abstract

Objective: Iliac crest autografts can compensate for severe mandibular atrophy before implant placement. However, the implant success in the augmented bone is not entirely predictable. Here we performed a retrospective cohort study to determine the success and related parameters of implants placed in augmented bone and pristine bone for up to 11 years. Material and Methods: We analyzed 18 patients where 72 implants were placed six months after iliac crest transplantation and 19 patients where 76 implants were placed in pristine bone. The primary endpoint was implant loss. Secondary endpoints were the implant success, peri-implant bone loss, and the clinical parameters related to peri-implantitis. Moreover, we evaluated the oral-health-related quality of life (OHIP). Results: Within a mean follow-up of 5.8 ± 2.2 and 7.6 ± 2.8 years, six but no implants were lost when placed in augmented and pristine bone, respectively. Among those implants remaining in situ, 58% and 68% were rated as implant success (*p* = 0.09). A total of 11% and 16% of the implants placed in the augmented and the pristine bone were identified as peri-implantitis (*p* = 0.08). Bone loss was similar in both groups, with a mean of 2.95 ± 1.72 mm and 2.44 ± 0.76 mm. The mean OHIP scores were 16.36 ± 13.76 and 8.78 ± 7.21 in the augmentation and the control group, respectively (*p* = 0.35). Conclusions: Implants placed in iliac crest autografts have a higher risk for implant loss and lower implant success rates compared to those placed in the pristine bone.

## 1. Introduction

Already when the term “osseointegration” was coined back in the 1970s, it was clear that this biological principle would revolutionize oral rehabilitation [1]. Back then, the implants were placed in edentulous patients with sufficient bone volume, while, today, challenging situations with severe mandibular atrophy are no longer excluded from implant therapy [2,3,4]. The two main strategies to compensate for the lack of volume are bone augmentation [5] and short implants [6]. Bone augmentation with autografts is challenging because it combines harvesting with transplantation surgery before the simultaneous or delayed placement of dental implants [4,7,8,9]. Considering that this procedure requires grafts consolidation with the local pristine bone and regaining soft-tissue integrity, the success of implants is presumably less predictable at sites following large augmentations than when placed in the pristine bone. However, recent developments regarding the osteotome technique (e.g., Magnetic Mallet) may provide an alternative to bone augmentation and serve as an additional device to improve primary implant stability [10].

Implant survival and success rates for implants placed in the mandible augmented with iliac crest are between 89% and 99% (observation period: 3.5–15 years) and between 86.9% and 100% (observation period: 1.5–5 years), respectively [2,11,12,13,14]. However, only a few studies [13,14] have adopted and reported on the success rate of implants placed in iliac crest augmented sites according to dedicated criteria [15,16]. Moreover, evidence has accumulated on the safety of intraoral augmentations performed with iliac-crest-bone harvesting with respect to its predictability [9,17], in contrast to bone resorption, which remains a major drawback of this technique [2,4,18]. Bone resorption may impair the outcome of peri-implant bone stability and, thus, the success of implants placed in the iliac crest bone [2].

Marginal bone loss is a surrogate parameter of implant success [16,19] and provides insights into how the augmented bone is maintained under functional loading and reflects inflammatory osteolysis that can escalate into peri-implantitis [20,21]. Together with the clinical parameters of bleeding on probing, probing depth, and plaque formation, the marginal bone loss indicates the health status of the peri-implant tissue. Previous studies on implants placed in mandible augmented with iliac crest showed a mean marginal bone loss of 0.6 and 1.8 mm after 7 and 5 years, respectively [2,11]. Besides bone resorption, donor site morbidity (e.g., pain, functional disorder, or sensory disturbance) is another drawback of iliac crest bone augmentation [22], which, as part of the overall treatment, may affect the patients’ quality of life (QoL). 

Patient-reported outcome measures (PROMs) are gaining attention in implant research involving the impact of bone augmentation. In a longitudinal study on post-menopausal women receiving dental implants with bone augmentation, the oral-health-related QoL (OHRQoL) improved continuously from the pretreatment to the 9-month assessment [23]. QoL has been reported differently following extra-oral bone harvesting. These patients demonstrated a lower health-related QoL compared to intra-oral donor sites immediately after graft harvesting [24]. A favorable outcome of OHRQoL has been indicated after a mean of 8 years following iliac-bone harvesting [25]. OHIP might thus be considered suitable to compare the OHRQoL after prosthetic rehabilitation of implants placed in the augmented and pristine bone.

Even though knowledge on the use of iliac crest autografts to augment the atrophic mandible is accumulating, there is always a demand to share clinical outcomes after years of dental implants in function. Therefore, the present study not only reports on how dental implants performed in iliac-crest-augmented bone; the presents study also shows a respective patient cohort where implants were placed in the non-augmented pristine bone with a mean follow-up of around six years. 

## 2. Material and Methods

### 2.1. Study Population

The local ethical committee of the Medical University of Vienna approved this clinical cross-sectional follow-up study (EK No. 1600/2014). The study is reported following the STROBE guidelines. The records of all patients, who received dental implants at the University Clinic of Dentistry, Medical University of Vienna between 2001 and 2013, were screened on the following eligibility criteria: (1) interforaminal bone augmentation with onlay graft from the iliac crest, (2) placement of four interforaminal implants, (3) presence of pre/post-implantation and at least one follow-up panoramic X-ray, (4) severely resorbed mandible [26], (5) follow-up > 1 year, (6) no previous horizontal osteotomy procedure to broaden a narrow ridge (Class IV; [27]), and (7) no severe systemic diseases (i.e., ASA ≥ 3). Smoking patients (*n* = 6) were included in the present analysis; among those were four heavy (≥10 cigarettes/day) and two light (<10 cigarettes/day) smokers. Patients who did not fulfill the criteria were excluded from this study. A final convenience sample of 39 patients (156 implants) could be enrolled in the present study consisting of 20 patients (80 implants) in the augmentation group and 19 patients (76 implants) in the corresponding control group selected based on the class of resorption [26] and time of implant placement. 

### 2.2. Clinical Treatment

**Figure 1:**
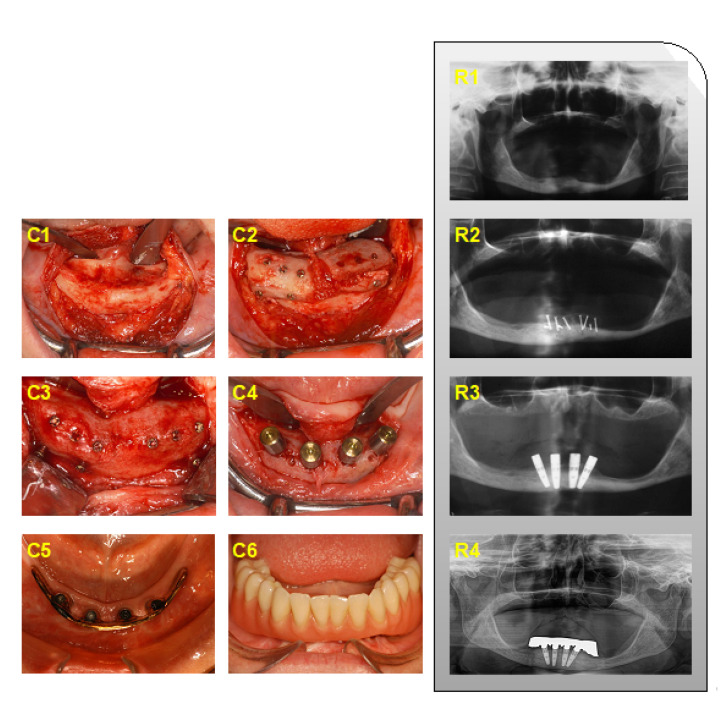
Clinical (**C1**–**C6**) and radiographic (**R1**–**R4**) presentation of iliac crest bone augmentation in the interforaminal region. (**C1**) Intraoperative situs; (**C2**) fixation of corticocancellous bone blocks; (**C3**) removal of osteosynthesis screws after healing; (**C4**) implant placement, (**C5**,**C6**) at the time of prosthetic reconstruction with; (**C5**) suprastructure with a milled bar; and (**C6**) final prosthesis in situ. (**R1**) Preoperative situation, (**R2**) after iliac crest bone augmentation, (**R3**) after implant placement, and (**R4**) follow-up after 11 years.

**Figure 2:**
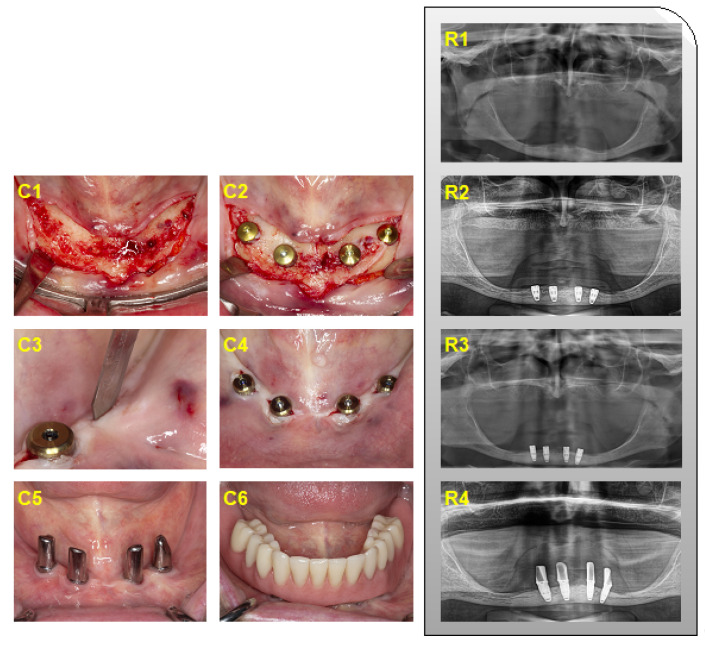
Clinical (**C1**–**C6**) and radiographic (**R1**–**R4**) presentation of interforaminal implant placement in pristine bone. (**C1**) Intraoperative situs, (**C2**) implants connected to cover screws, (**C3**) uncovering of implants, (**C4**) implants connected to healing abutments, (**C5**,**C6**) at the time of prosthetic reconstruction; (**C5**) implants connected to telescopic crowns, and (**C6**) final prosthetic outcome. (**R1**) Preoperative situation, (**R2**) after implant placement, (**R3**) after uncovering of the implants, and (**R4**) implant loading for 1.5 years.

The augmentation procedure with onlay grafts was performed under general anesthesia. All patients received intravenous antibiotics 30 min prior to surgery. The skin incision was performed 2 cm dorsal to the Spina iliaca anterior superior after local infiltration with ropivacaine HCL (Naropin^®^, Aspen Pharma Trading Ltd., Dublin, Ireland). The iliac crest was depicted following sharp preparation under the protection of the N. cutaneus femoris lateralis and the cutaneous branch of the N. iliohypogastricus. Over-sized corticocancellous bone blocks were harvested by using an oscillating saw from the superior–anterior aspect of the iliac crest. The defect at the harvesting site was covered with oxidized regenerated cellulose (Tabotamp^®^, Ethicon^®^, Johnson & Johnson Medical GmbH, Norderstedt, Germany) before layered suturing. A redon drain was applied for 24 h. Simultaneously, recipient sites of the interforaminal region were prepared by raising a mucoperiosteal flap and cortical perforation of the recipient bed. The appositional bone blocks were fixed by using osteosynthesis screws (KLS Martin, Tuttlingen, Germany) per segment. Wound closure was achieved by releasing incision of the mucoperiosteal flap and mattress sutures. A provisional prosthesis was not allowed within the first 8 weeks after onlay grafting. After a healing period of six months, four implants (internal connection: Replace Select Tapered/Straight/Groovy; or external connection: Brånemark System Mk III, Nobel Biocare^TM^, Kloten, Switzerland) were placed in the interforaminal region according to the manufacturer’s protocol. Implants were connected to healing abutments (one-stage surgery) or uncovered six to eight weeks later in a two-stage surgery. The prosthetic rehabilitation was performed with a removable denture retained either by a milled bar or telescopic crowns (Figure 1). Implant placement and prosthetic rehabilitation were also performed in the interforaminal region without augmentative procedures (Figure 2).

### 2.3. Clinical Parameters

Implants were monitored directly after delivery of the implant-retained denture and thereafter at every follow-up visit. Clinical examination involved percussion and manual manipulation. Bleeding on probing (BoP), plaque, and pocket probing depth (PPD) were assessed by using a periodontal probe. Marginal bone loss (MBL) at the mesial and distal site of each implant was evaluated on panoramic X-rays at ≥3 different time points: T_0_ (time of implant placement), T_1_ (delivery of implant-supported prosthesis), and T_1+n_ (any follow-up thereafter). The measurements were performed on either analog panoramic radiographs with a precision slide jaw caliper with a maximum resolution of 0.01 mm (Zuercher Model, Planer, Austria) [28] or on digital panoramic radiographs with dedicated software (Sidexis XG^®^, Sirona Dental, Bensheim, Germany) on a diagnostic monitor (RadiForce^TM^ G22, EIZO, Hakusan, Japan). 

### 2.4. Marginal Bone Level

The ratio marginal bone level to implant–abutment interface was assessed at the time of follow-up (T_1+n_) and compared to T_0_; the distance between baseline and follow-up was considered as peri-implant bone loss per site. The actual bone loss was obtained after adjusting the radiologic magnification by calculating the ratio between the radiographic implant and the real implant size. The bone level at the time of implant placement was considered as the baseline, which does not necessarily correspond with the implant shoulder. Positive values signify bone levels above the implant shoulder. Measurements on radiographs were performed twice by the same examiner with six weeks in between (ICC: 0.981). Finally, the mean value of both measurements was considered for further analysis. Implant success was defined according to the criteria of Albrektsson et al. [15], respectively the ICOI Pisa Consensus implant quality of health scale [16]. Peri-implantitis was defined as the presence of BoP and/or suppuration, PPD > 5 mm, and radiologic bone loss [20,29]. At the last follow-up, patients were invited to fill in an Oral Health Impact Profile (OHIP-G) questionnaire [30].

### 2.5. Statistical Analysis

Implant survival is represented by Kaplan–Meier curves. For inductive statistics on variables on the location level (marginal bone loss, pocket probing depth), we used linear mixed models with nested random effects of patient ID, implant ID, and time from implantation and treatment group, as well as other potential confounders, as fixed effects. Note that this approach takes the clustered structure of the data into account. Variables on the implant level used similar models but without a random implant effect. All of these models were fitted by using the package lme4 [31]. Tests within those models were based on Wald-tests and F-distributions approximated by the Kenward and Roger approach [32]. For induction on success, we used a generalized mixed logit model, including the same fixed and random effects. Tests for this model were based on the parametric bootstrap [33]. The effect of augmentation on implant survival was tested by Firth’s method for rare events [34]. To test for differences in OHIP-score on the patient level, we used a permutation test based on the t-statistic [35]. All computations were performed by using R version 4.0.3 (R Core Team 2015, Vienna, Austria). Statistical Graphics were created by using the package ggplot2 [36].

## 3. Results

### 3.1. Patient Characteristics

Between 2001 and 2013, a total of 20 patients were rehabilitated with dental implants inserted in the interforaminal region after augmentation with onlay grafts from the iliac crest. Two patients were not available for follow-up and were excluded from the present analysis. The final sample consisted of 18 and 19 patients with a mean age (years) of 53.4 ± 9.8 and 66.5 ± 8.9 in the augmentation and in the control group at the time of implant placement, respectively. All patients, except for one, were female. The longest observation periods were 11.0 and 11.5 years in the augmentation and in the control group, respectively. Management of the soft-tissue situation was performed in three cases by vestibuloplasty in the augmentation and in one case by a free gingival graft in the control group, respectively. The distribution of the implant–abutment interface was 14% (external)/35% (internal) and 24% (external)/27% (internal) in the augmentation and in the control group, respectively. 

### 3.2. Implants Survival and Implants Success

**Figure 3:**
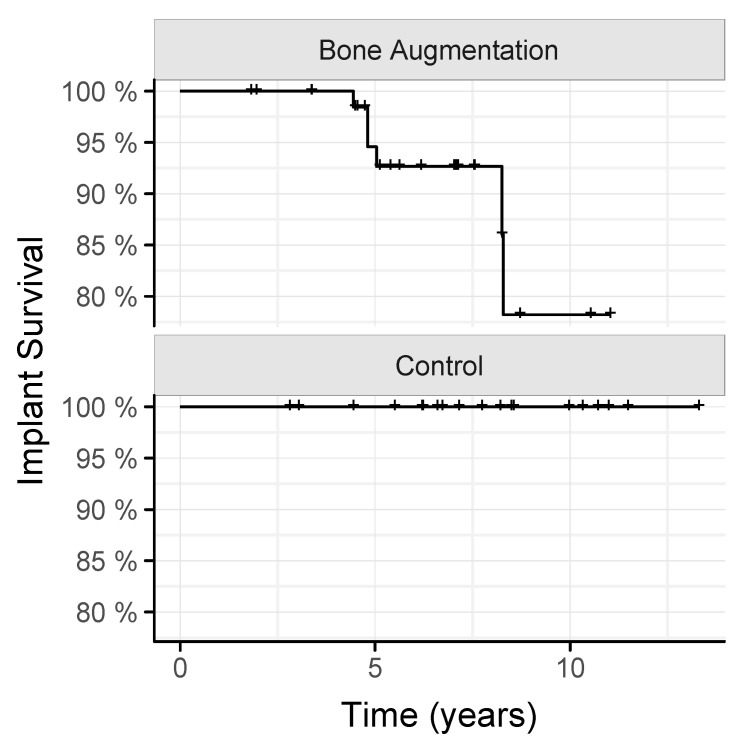
Kaplan–Meier survival curve of implants placed in iliac crest augmentations and pristine bone (control).

To understand how the augmentation with the iliac crest affects the survival and success of dental implants, we followed up these patients for a mean of 5.8 ± 2.2 years, and patients with implants placed in pristine bone serving as a control for a mean of 7.6 ± 2.8 years. We report here that six implants of three patients were lost to follow-up among the 72 implants placed in the iliac-crest-augmented bone. No implants were lost when placed in the pristine bone (*p* = 0.085; Figure 3). Among the remaining implants, a total of 58% and 68% of the implants in the augmentation and the control group were rated as implant success (*p* = 0.09). Based on another calculation, 86% and 95% of the implants in the augmentation and the control group were rated as satisfactory survival. Thus, there is a trend that the performance of implants placed in the iliac crest augmentation is impaired compared to those placed in the pristine bone.

### 3.3. Peri-Implant Health

The clinical parameters were recorded for the evaluation of peri-implant health: BoP (%) measured at the implant-level was positive in 48.61 ± 50.29 and 52.63 ± 50.26 in the augmentation and the control group, respectively (*p* = 0.66). When evaluating the presence of plaque (%) at the implant-level, we had 66.67 ± 44.88 and 78.95 ± 41.04 in the augmentation and the control group, respectively (*p* = 0.59). The mean PPD was 3.58 ± 1.28 mm and 3.62 ± 1.62 mm in the augmentation and the control group, respectively (*p* = 0.94). Peri-implantitis was diagnosed in 11.11% and 15.79% of all the implants in the augmentation and the control group, respectively (*p* = 0.08). Suppuration was only observed in the augmentation group and present in five out of the six failing implants. 

### 3.4. Marginal Bone Loss

**Figure 4:**
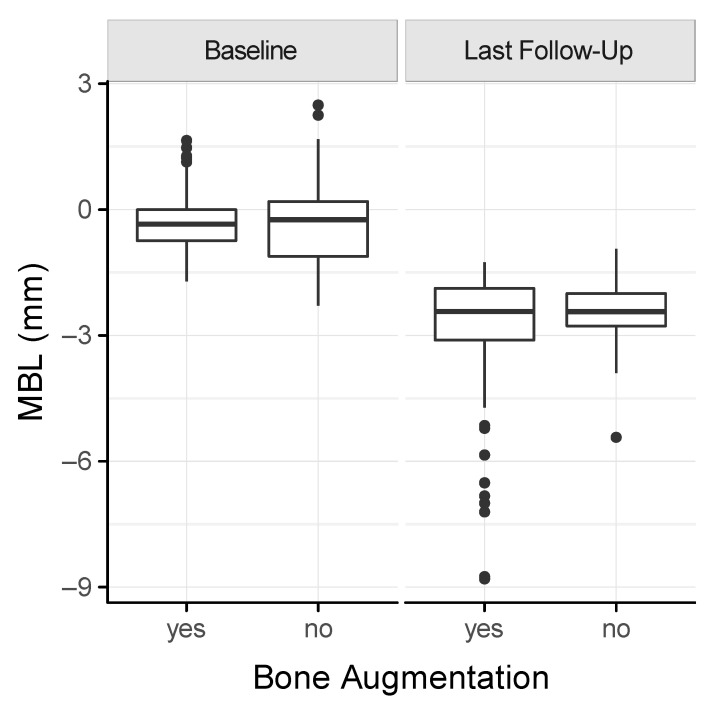
Box plot of the marginal bone loss (MBL) at the time of implant placement (baseline) and the last follow-up.

**Figure 5:**
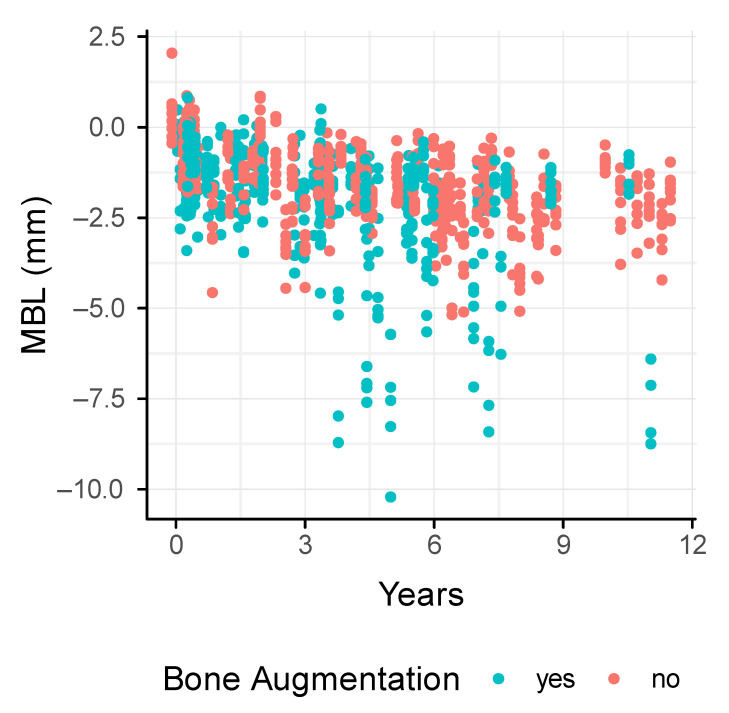
Trend of the marginal bone loss (MBL) of each implant over time up to 11 years of follow-up between the augmentation and the control group.

We next determined the marginal bone loss around the dental implants based on panoramic radiographs. The mean MBL was 2.95 ± 1.72 mm and 2.44 ± 0.76 mm in the augmentation and the control group, respectively (Figure 4). The MBL increased in both groups over time (Figure 5). There was a tendency towards a higher MBL in the augmentation compared to the control group, particularly after 3 years (*p* = 0.1). In addition, smokers revealed a mean of 4.66 ± 2.58 mm and 2.92 ± 0.44 mm in augmented and pristine bone. Non-smokers showed a mean of 2.46 ± 0.96 mm and 2.38 ± 0.78 mm in the augmentation and the control group, respectively. Even though underpowered, the MBL of smokers after iliac crest transplantation is almost twice as high compared to smokers in the control group. 

### 3.5. Oral-Health Impact Profile (OHIP)

Finally, we evaluated the score that provides a self-rating patient-centered instrument designed to assess the priorities of care. At the time of recall, 14 and 9 patients in the augmentation and control group completed the OHIP questionnaire. The OHIP summary scores were in mean 16.36 ± 13.76 and 8.78 ± 7.21 in the augmentation and the control group (*p* = 0.35). These scores thus complement the overall picture that implant placement in iliac-crest-augmented bone in the esthetic region is more demanding than when implants are placed in the pristine bone. 

## 4. Discussion

This retrospective study is the first to compare the survival and success of interforaminal implants in iliac crest onlay grafts with a control group in a single institution. Our research was based on the clinical demand for evidence when it comes to clinical outcomes of dental implants placed in iliac-crest-augmented bone after years in function. We, therefore, followed 18 and 19 patients with and without iliac crest augmentation for up to 11 years. Implant loss was restricted to the augmentation group corresponding to an absolute survival rate of 91.7%. When considering the implants remaining in situ, implant success was 58% and 68% of the augmentation and the control group, respectively, suggesting that implant placement in the interforaminal region after iliac crest augmentation is compromised compared to pristine bone. These findings are relevant, as they add to the existing evidence that implant placement in iliac crest augmented bone is feasible but requires special attention in the recall, as it is associated with a higher risk for implant loss and lower implant success. 

If we relate our findings to those of other long-term studies, similar implant survival rates have been provided for implants placed in iliac onlay grafts of the mandible varying between 91.6% (follow-up: 26 years) [17] and 98.7% (follow-up: 5 and 15 years, respectively) [2,12]. A drop of 10% survival rate from 92% to 82% of maxillary and mandibular implants in iliac-crest-augmented bone after 21 years was also reported [9]. The implant success rate provides a more reliable prediction of an ideal implant outcome [37] and thus facilitates the comparison of peri-implant health. Two studies reported on the success rate of implants placed in iliac-augmented bone [15,16]: 86.9% (mandible) and 96.6% (maxilla/mandible) after a follow-up of 1.5 (median) and 4.2 years (mean), respectively [13,14]. In our study, the implant success rate was considerably lower for iliac grafted sites, and this may be explained by the longer observation period, a different anatomic region, the study population, and the type of prosthetic restoration. Furthermore, a history of periodontitis linked to peri-implantitis [38,39] could not be retrieved from the patients’ records, as almost all were referred edentulous to our institution. 

The marginal bone loss has a strong impact on the implant success rate, and, hence, it was high in the present study, with a mean of 2.9 mm in the augmentation and 2.4 mm in the control group, respectively. If related to others, a mean of 0.6 to 1.8 mm has been indicated for mandibular implants in iliac grafted sites after a follow-up of up to 6 years [2,11,13]. The increased MBL in the present study requires a closer reflection: First, the only type of prosthetic restoration was a removable denture, which has demonstrated an odds ratio of 2.6 for higher MBL [40]. Second, we included a total of six smoking patients, who demonstrated a mean MBL of 4.08 ± 2.56 mm compared to 2.42 ± 0.86 mm of the non-smoking collective. Smoking patients with augmentation were prone to an almost doubled mean MBL of 4.67 ± 2.58 mm compared to 2.38 ± 0.78 mm of non-smoking patients without augmentation, as is consistent with findings from others [8]. Third, 38% of all implants were configured with an external connection; however, recent systematic reviews and a meta-analysis demonstrated lower MBL for internal connections [41,42]. In the present study, the MBL was higher than 2 mm [16,19], suggesting that a more stringent supportive peri-implant therapy (SPT) is indicated. 

In our study, six implants in three patients were lost late after a mean of 6 years in the augmentation group, due to peri-implantitis. One patient experienced multiple visits of SPT and resective implantoplasty to stop progressive bone loss [43]. In contrast, the other two patients were not available for SPT, and no surgical treatment was performed prior to the explantation of the implants. However, all remaining implants of these patients could be kept, in two patients following resective implantoplasty and adaptation of the removable denture. It should be noted that two of these patients were heavy smokers, and smoking is associated with a higher risk of implant failure [44]. It remains unclear at this point whether the loss of the implants in the augmentation group can be attributed to extensive bone remodeling and thus graft resorption, possibly reaching 87% for iliac crest bone grafts in the mandible after 6 years [45]. Furthermore, an impact of this graft resorption on peri-implant bone loss could not be observed in the present study, as the MBL of the augmentation and the control group was rather similar. Our clinical parameters of BoP and plaque were unfavorable; however, peri-implantitis was indicated with 11–16%, which is acceptable after a period of 5–10 years [46]. Considering the limited capability of BoP to identify peri-implantitis [46,47], the data support the importance of radiological monitoring in implant dentistry. 

The OHRQoL scores reported here were comparable to those reported by others following iliac crest augmentation [25,48]. A mean OHIP score of 8.4 has been reported after 7.8 years following iliac-crest harvesting [25]. In the present study, patients of the augmentation group exhibited a slightly higher OHIP score compared to the control after several years of the implant in function, which cannot be explained by a previous donor site morbidity. The quality of life was excellent/very good in 82% of the patients following augmentation, even after suffering from donor site morbidity [49,50]. Donor-site morbidity is nevertheless a drawback when it comes to large augmentation with autografts [51]. We are aware of the limitation of the OHIP, as this score is restricted to oral-health-related parameters. For instance, others have additionally used the SF-36 score to measure the general HRQoL, which may consider the morbidity caused by extra-oral autograft harvesting [24]. Thus, even though we have not considered donor-site morbidity, we could identify the group of patients who underwent iliac crest augmentation and implant placement to have a comparable oral-health-related quality of life compared to those patients where implants were placed in the pristine bone. 

This study has limitations. First, the sample of both cohorts was all-female, except for one patient; thus, this restricts the extrapolation of the results to the general population [17]. Still, a significant difference between gender and MBL was reported in a 10-year retrospective study. While female patients had a mean MBL of 2 mm (0.5–4 mm), male patients indicated a lower MBL of 1 mm (0.5–2 mm) [11]. Furthermore, implant failures occurred more often in female patients in onlay grafted maxillae after a mean of 11 years [52]. In this context, the retrospective character of the present study may be considered a limitation, as prospective studies would facilitate equal allocation of patients by gender. Second, the sample size is limited; however, it is based on the number of iliac onlay grafts performed at our institution matching the inclusion criteria, and therefore a sample size calculation was not conducted. This can be further explained by the decline of iliac-crest-harvesting procedures in favor of short implants to avoid augmentation-related complications. Third, whether changes occurred regarding the iliac bone density following loading of the implants cannot be elucidated from the present data. Two high-resolution CTs would be an ideal premise for this analysis. Future studies may therefore consider a multi-center approach to provide more data on the success of implants and the influence of gender in iliac-crest-augmented bone in a larger sample, as well as a CT-based analysis of morphological changes of transplanted bone after functional loading. 

## 5. Conclusions

Implants in iliac-crest-augmented sites of the anterior mandible are at higher risk for failure and have lower success rates compared to implants placed in the pristine bone, in a predominantly female cohort. Supportive peri-implant therapy is crucial, especially for smoking patients with iliac-crest-augmented bone, as they are prone to higher MBL compared to non-smokers. Despite the longer duration of treatment and possible donor-site morbidities, the quality of life can be improved in patients undergoing iliac crest augmentation in the long term. 

## Data Availability

Data supporting the reported findings are available from the corresponding authors upon reasonable request.
